# Color-dependent changes in humans during a verbal fluency task under colored light exposure assessed by SPA-fNIRS

**DOI:** 10.1038/s41598-021-88059-0

**Published:** 2021-05-06

**Authors:** Hamoon Zohdi, Rahel Egli, Daniel Guthruf, Felix Scholkmann, Ursula Wolf

**Affiliations:** 1 University of Bern, Institute of Complementary and Integrative Medicine, Fabrikstrasse 8, 3012 Bern, Switzerland; 2 Biomedical Optics Research Laboratory, Neonatology Research, Department of Neonatology, University Hospital Zurich, University of Zurich, 8091 Zurich, Switzerland

**Keywords:** Neuroscience, Cognitive neuroscience, Neuro-vascular interactions, Sensory processing, Visual system

## Abstract

Light evokes robust visual and nonvisual physiological and psychological effects in humans, such as emotional and behavioral responses, as well as changes in cognitive brain activity and performance. The aim of this study was to investigate how colored light exposure (CLE) and a verbal fluency task (VFT) interact and affect cerebral hemodynamics, oxygenation, and systemic physiology as determined by systemic physiology augmented functional near-infrared spectroscopy (SPA-fNIRS). 32 healthy adults (17 female, 15 male, age: 25.5 ± 4.3 years) were exposed to blue and red light for 9 min while performing a VFT. Before and after the CLE, subjects were in darkness. We found that this long-term CLE-VFT paradigm elicited distinct changes in the prefrontal cortex and in most systemic physiological parameters. The subjects’ performance depended significantly on the type of VFT and the sex of the subject. Compared to red light, blue evoked stronger responses in cerebral hemodynamics and oxygenation in the visual cortex. Color-dependent changes were evident in the recovery phase of several systemic physiological parameters. This study showed that the CLE has effects that endure at least 15 min after cessation of the CLE. This underlines the importance of considering the persistent influence of colored light on brain function, cognition, and systemic physiology in everyday life.

## Introduction

Light is essential not only for vision but also for the regulation of sleep and wakefulness, neurobehavioral, and neuroendocrine functions^1–7^. Among these nonvisual (i.e., non-image-forming) functions, light exposure has a direct impact on alertness and cognitive abilities^8,9^. Cognition is modulated by circadian rhythms and the nonvisual effects of light^10^. Short-wavelength (e.g. blue) monochromatic light affects the circadian rhythms of cognitive functions mediated by a melanopsin-based photoreceptor system^11^. One main class includes the intrinsically photosensitive retinal ganglion cells (ipRGCs), which are most sensitive to blue light (~ 460–480 nm)^1,12^. Thus, exposure to blue light influences many physiological functions, and it is applied to treat circadian and sleep dysfunctions, seasonal affective disorder as well as to boost alertness, help cognitive function, and elevate mood^13–15^.

Whether exposure to colored light has an effect on cognition in humans is a current research question. Neuroimaging studies devoted to this topic employed electroencephalography (EEG)^11,16,17^, positron emission tomography (PET)^18^, and functional magnetic resonance imaging (fMRI)^1,12,19–22^. These human studies showed that light exposure affects cortical areas involved in the cognitive process and improves alertness and cognitive performance. In particular, the influence of brain responses (e.g., functional connectivity between hypothalamus and amygdala) and the cognitive performance were higher during blue light in comparison with longer wavelengths^1,20,22^.

Among numerous cognitive tasks to assess cognitive functioning, the verbal fluency task (VFT) is a common neuropsychological test, which challenges the cognitive functioning during the arduous retrieval and verbal articulation of words^23^. In this test, subjects are instructed to produce as many words as possible within a restricted time, beginning with a certain letter (phonemic task) and/or belonging to a certain category of words (semantic task). The performance of these tasks is attributed to indicators of vocabulary size, lexical access speed, inhibition ability, and updating^24^. The performance of the test is mainly mediated by temporal, frontal, and parietal cortices^25–27^.

These cortices can easily be investigated by functional near-infrared spectroscopy (fNIRS), a neuroimaging technique, which measures changes in cerebral tissue hemodynamics and oxygenation related to alterations in neuronal activity^28–30^. One of the key advantages of fNIRS compared to fMRI is the relative insensitivity of this method to speech-related movement artefacts^31–33^. fNIRS is able to non-invasively and continuously determine concentrations of oxygenated ([O_2_Hb]) and deoxygenated ([HHb]) hemoglobin of the cortical layers of the human brain^34,35^. It is known that cognitive activation leads to an increase in oxygen consumption, which is accompanied by an increase in cerebral blood flow (CBF) and total hemoglobin concentration ([tHb]) due to neurovascular coupling, resulting in an increase in [O_2_Hb] with a concurrent decrease in [HHb]^28,36,37^. Since [O_2_Hb] and [HHb] are affected by factors not related to brain activity, i.e., changes in the systemic physiology, these factors need to be measured concurrently^38–40^. Therefore, it is crucial to employ the systemic physiology augmented (SPA) fNIRS approach, which relies on the measurement of brain activity with fNIRS along with the assessment of changes in systemic physiology—an approach our research group has been investigating for several years^38,39,41,42^. SPA-fNIRS is an ideal approach to avoid misinterpretations of fNIRS signals^43^ as well as for a complete understanding of how the whole body reacts to task/stimulus paradigms.

The main goal of this study was to investigate by SPA-fNIRS how colored light exposure (CLE, red and blue light) and a VFT interact and affect cerebral hemodynamics and oxygenation, as well as systemic physiology. The findings of the current study are expected to facilitate a better understanding of the effect of colored light on cognition and behavior. The results have a broad range of implications for daily human life.

## Subjects and methods

### Subjects

32 healthy subjects (17 female, 15 male, age 25.5 ± 4.3 years, range 19–45 years) participated in this study after they signed written informed consent. Subjects were all right-handed, non-smokers, medication-free, and with high education level (i.e., university students or university degree). They were asked to refrain from consuming caffeine and eating two hours prior to the experiment. The study was conducted in accordance with the World Medical Association Declaration of Helsinki, and the protocol and all methods were approved by the Ethics Committee of the canton of Bern (Project identifier: COLOR10; Basec-Nr. 2016-00674).

### Experimental protocol

**Figure 1:**
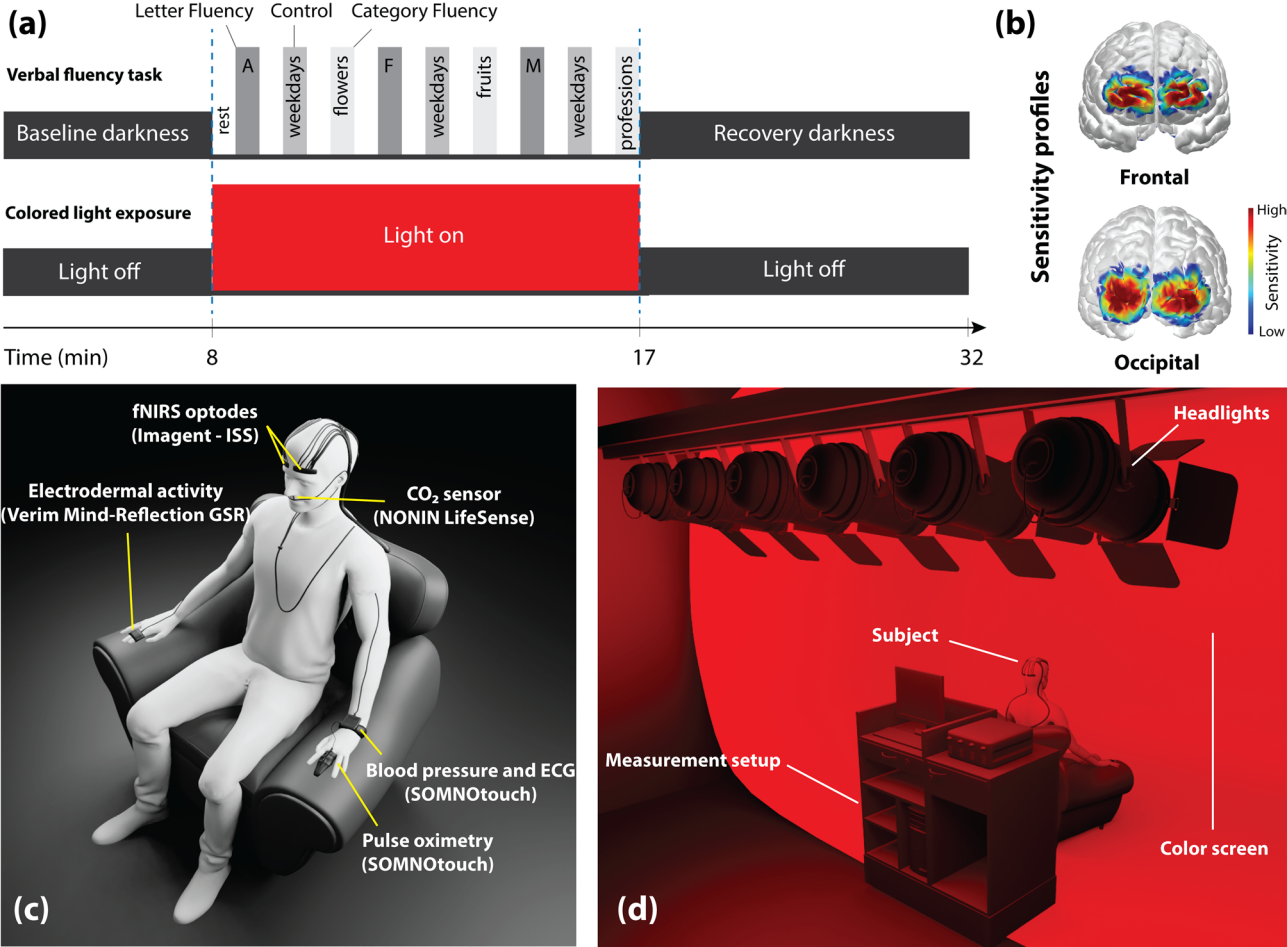
(**a**) Schematic illustration of the experimental protocol, including the VFT and CLE. (**b**) Sensitivity profile of ISS-sensors on the brain. The sensitivity profile shows which regions of the brain are measured with fNIRS. The higher the value, the more contribution of the fNIRS signal from the cerebral cortex layer. (**c**) Visualization of the placement of devices/sensors on the subject. (**d**) Experimental setup with the position of the subject and the color screen.

The subjects were asked to sit upright in a comfortable reclining chair in a dark room, while a white wall was in front of them (distance from the subject to the wall: 160 ± 5 cm). Following a randomized crossover design, each subject was exposed to two different light colors (red and blue). The spectrum of the light sources was measured by an Ocean Optics spectrometer and showed a peak wavelength of ~ 640 nm (full width at half maximum FWHM ~ 20 nm) for red and ~ 450 nm (FWHM ~ 20 nm) for blue. The illumination was adjusted to 120 lx (Digital Lux Meter, DT-1308, ATP) at the eye for both colors. The subjects were exposed to the colored light for 9 min on two different days but at the same time of day to minimize circadian variability of the responses. Before (baseline, 8 min) and after (recovery, 15 min) CLE, subjects were in darkness. The VFT, which contained three sessions, was carried out during CLE. Each session comprised three different trials in which the subjects had to produce as many words as possible within 30 s: (i) letter fluency task: producing nouns with a given letter (A, F, or M); (ii) control task: reciting weekdays in a consecutive manner; (iii) category fluency task: producing words from a specified category (flowers, fruits, or professions). The instruction of these tasks was given to the subjects by audiotaped voices. Figure 1a shows the schematic representation of the experimental VFT protocol used in this study. The order of the type of the trials (letter fluency, control, and category fluency) was fixed across all measurements, but the specific letters and categories were used in randomized order. Thus, each measurement period consisted of 9 trials of 30 s duration. The 1st, 4th, and 7th trials were letter fluency tasks with the letters A, F, or M in randomized order. The 2nd, 5th, and 8th trials were control tasks, where the subjects recited weekdays. The 3rd, 6th, and 9th trials were category fluency tasks with randomized categories. Each trial was followed by a resting phase of 30 s, where the subjects were asked to relax and stop mental engagement regarding previous tasks. The total duration of the VFT was 9 min and the period of the CLE was adjusted to this period. They were asked to keep their eyes open throughout the entire measurement and to move their head or body as little as possible during the measurement to avoid movement artifacts.

### Measurement setup

With a multi-channel frequency-domain near-infrared spectroscopy (FD-NIRS) system (Imagent, ISS, Inc., Champaign, IL, USA), employing a multi-distance approach, changes of [O_2_Hb], [HHb], [tHb] and tissue oxygen saturation (StO_2_) were measured at a sampling rate of 2.5 Hz on the prefrontal cortex (PFC) and visual cortex (VC). The ISS optodes were positioned bilaterally over the PFC (left: Fp1 and right: Fp2) and the VC (left: O1 and right: O2), according to the international 10–20 system. The sensitivity profile of the optodes on the brain is shown in Fig. 1b. A detailed description of the FD-NIRS data acquisition and imaging instrumentation can be found in our previous studies^44,45^.

Heart rate (HR) was measured with a SOMNOtouch NIBP device (SOMNOmedics GmbH, Randersacker, Germany) with a sampling rate of 4 Hz. SOMNOtouch calculated the HR from the ECG data by calculating the R-R intervals. This device also measured and determined the following parameters at a sampling rate of 1 Hz: Mean arterial pressure (MAP), pulse pressure (PP), arterial oxygen saturation (SpO_2_), high-frequency (HF; 0.15–0.4 Hz), and low-frequency (LF; 0.04–0.15 Hz) component of the heart rate variability (HRV).

A NONIN LifeSense (NONIN Medical, Plymouth, MN, USA) was used to non-invasively measure end-tidal carbon dioxide (P_ET_CO_2_) and respiration rate (RR). Data were recorded at a sampling rate of 1 Hz.

An electrodermal activity measurement system (Verim Mind-Reflection GSR, Poland) was employed to determine the skin conductance (SC). Skin conductance level (SCL) and integrated skin conductance response (ISCR) were measured at 8 Hz sampling rate.

To quantify the coupling between HR and RR, the pulse-respiration quotient (PRQ) was calculated (PRQ = HR/RR)^46^.

All data were recorded simultaneously. The position of devices and sensors on the subject is shown in Fig. 1c. Moreover, Fig. 1d presents the experimental setup with the position of the subject and the color screen. The 3D human model was constructed using the Blender 3D interface (http://www.blender.org, version 2.82).

### Signal processing and statistical analysis

The data set of one subject was excluded from data analysis since the subject was not a German native speaker, which might have affected the VFT performance. All signal processing and statistical analysis were performed in MATLAB (R2017a, MathWorks, Inc., MA, USA).

#### Cerebral oxygenation and hemodynamics

Movement artefacts in fNIRS signals were detected and removed by the in-house developed movement artefact removal algorithm (MARA) based on moving standard deviation and piecewise-interpolation^47^. For 96.7% of the signal time series, no processing with MARA was required. When MARA was applied, it was ensured the overall trend of the time-series processed was not altered. To further remove high-frequency noise, signals were low pass filtered using a robust 2nd-degree polynomial moving average (RLOESS) filter with a span of 3 min. Signals from the left and right PFC and VC were subsequently averaged to obtain signals for the whole PFC and VC, respectively.

#### Systemic physiological parameters

All other biosignals, except the SC, were also denoised by the RLOESS method with a window length of 3 min. The SC data were processed with Ledalab toolbox (http://www.ledalab.de)^48,49^. This MATLAB toolbox is able to extract the phasic (high frequency) and tonic (low frequency) SC components by continuous decomposition analysis. In this study, the tonic component of the SC, known as skin conductance level (SCL), and the ISCR, which involved the integration (i.e., area under the curve) of the phasic driver signal, were used for the signal processing and statistical analysis.

#### Statistical analysis

All bio-signals were segmented into ten parts (3 min each), and the median of each segment was calculated. Two segments were assigned to the baseline phase (2–8 min, time points (TP) 1–2), three to the CLE (TP 3–5), and five to the recovery phase (TP 6–10). The median of each segment was then normalized to the last TP of the baseline phase (TP2). Subsequently, to analyse and visualize the changes of the parameters at the group-level, the median and the standard error of the median (SEM) of each segment among all subjects were calculated. Additionally, a Wilcoxon signed-rank test for each TP in comparison with the last TP of the baseline (TP2) was calculated and a false discovery rate (FDR) correction was subsequently applied to the *p*-values in order to correct for the multiple comparison situation. In order to compare the effects of the two types of colored light (red vs. blue) on the changes of the bio-signals during the task/stimulation and recovery phases, each time point of red light was compared with the same time point in blue light using the Wilcoxon signed-rank test. Moreover, the number of correct words generated by each subject during the task period was determined as the subjects’ task performance. Word repetitions and proper names of people and places were excluded from the data analysis. Synonyms and direct hypernyms/hyponyms were counted as one word. The effects of sex and type of cognitive task on subjects’ performance were also investigated by the *t*-test and paired Wilcoxon signed-rank test, respectively. For the behavioral data, a 2 × 2 × 2 analysis of variance (ANOVA) was applied with JASP (jasp-stats.org, version 0.11.0.0) to test for the main effects of sex, CLE, and VFT as well as for interaction effects. Investigating the effects of sex as well as the specific type of VFT on cerebral and physiological parameters is interesting, but it is beyond the scope of this article and will be analyzed in-depth in a future investigation.

## Results

### Task performance

**Figure 2:**
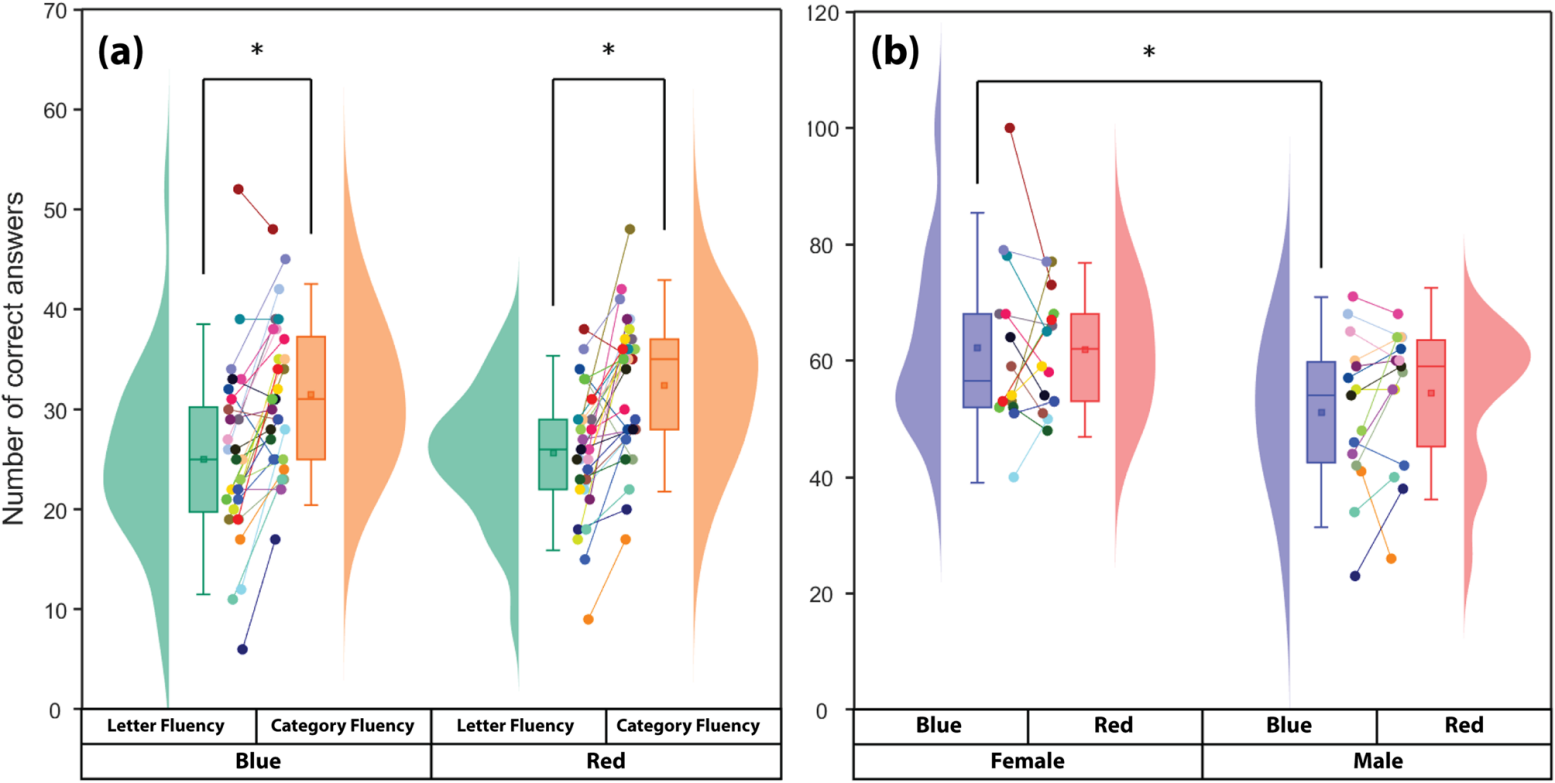
(**a**) Subjects’ performance in the category and letter versions of the VFT during CLE. Asterisks show a significant difference between tasks (*p* < 0.05, Wilcoxon signed-rank test). (**b**) Effects of sex on the task performance of subjects who are exposed to two lighting conditions (blue vs. red). The asterisk shows a significant difference between male and female subjects during blue light exposure (*p* < 0.05, two-sample *t*-test). The same color data points and lines belong to an individual subject.

Subjects were more successful in the category version of the VFT compared to the letter fluency version (blue: *p* < 0.001; effect size (Cohen’s d): *d* = 0.8, red: *p* < 0.001; *d* = 1.0). No significant difference in the performance of both tasks was found between blue and red light. In more detail, subjects produced 25.0 ± 9.0 (mean ± SD) nouns in the letter fluency during the blue light condition, while they reached a number of 25.6 ± 6.5 nouns during the red light. In the category fluency, subjects produced 31.5 ± 7.4 and 32.3 ± 7.1 nouns during blue and red light exposure, respectively (Fig. 2a).

### Sex effects

The total number of correct responses during the blue light condition for the female and male subjects was 62.2 ± 15.5 and 51.1 ± 13.2, respectively, whereas they produced 61.9 ± 10.0 (female) and 54.3 ± 12.1 (male) correct words during red light exposure. Females were generally better VFT performers compared to males. Although the difference was significant during the blue light condition (*p* < 0.05; *d* = 0.8) (Fig. 2b), no significant interaction effect was found for “Sex × Colored light” analyzed by ANOVA (Table 1).

**Table 1:** Summary of the ANOVA for subjects’ performance. Values in bold indicate statistical significance at the *p* < 0.05 level.

	VFT correct responses	
	F-statistic	*p*-value
**Main effects**		
Sex (S)	**11.906**	** < 0.001**
Colored light (C)	0.278	0.599
VFT tasks (T)	**23.913**	** < 0.001**
**Interaction effects**		
S × C	0.435	0.511
S × T	0.064	0.800
C × T	0.005	0.942
S × C × T	0.222	0.639

### Changes during the colored light exposure and verbal fluency tasks

**Figure 3:**
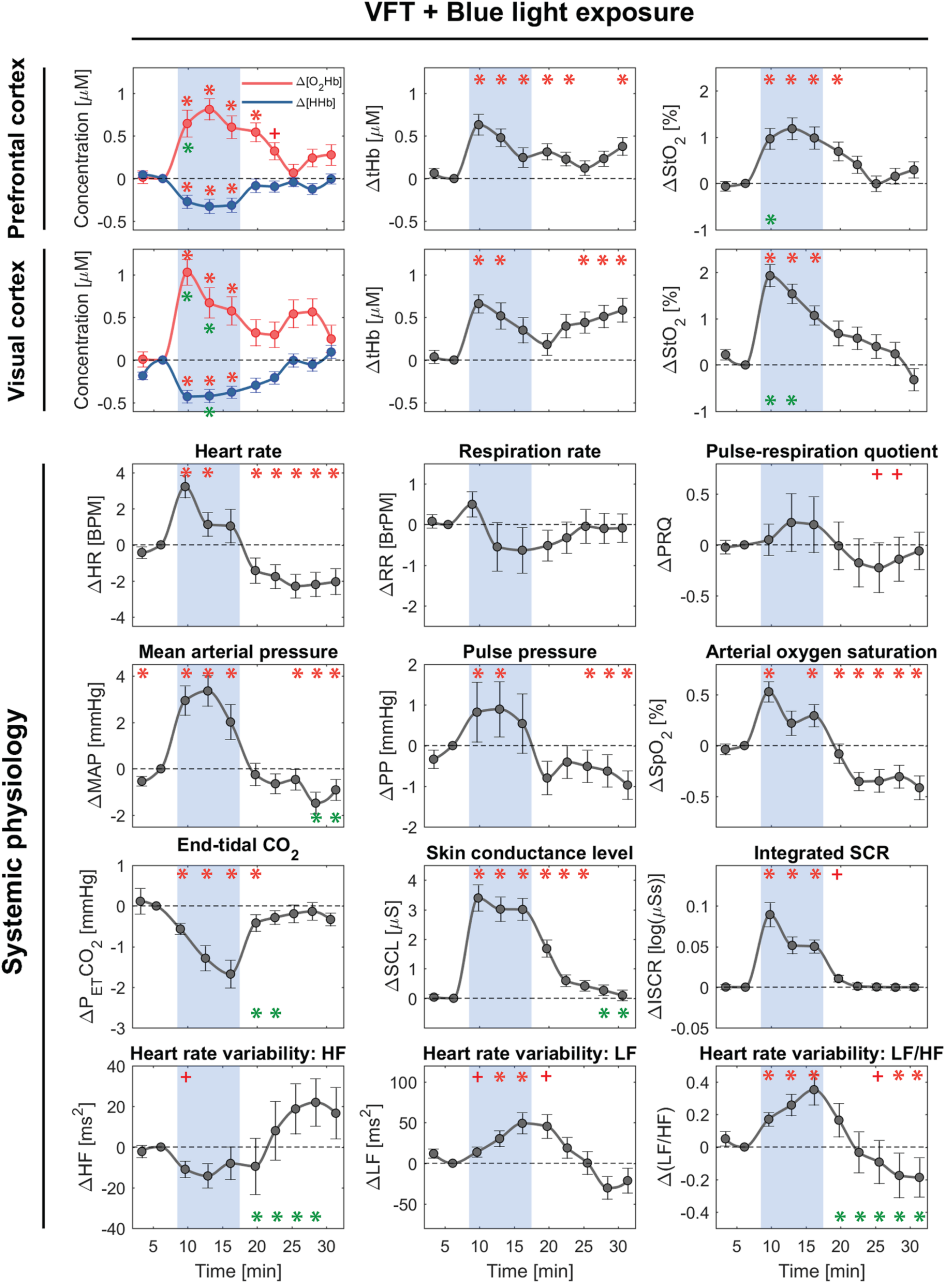
Block-averaged (group-level) changes in cerebral hemodynamics/oxygenation and systemic physiology (median ± SEM) evoked by blue light exposure and VFT. The blue shaded areas represent time intervals during the task/stimulation period. The time series are sub-divided into ten periods (3 min each). Then, they are normalized to the last time period of the baseline (TP2). Red symbols indicate a significant change of the marked time point with respect to baseline (asterisk *: proved by both FDR-corrected and uncorrected *p* values; plus + : only uncorrected *p* value, *p* < 0.05, Wilcoxon signed-rank test). Green asterisks present a color-difference (blue vs. red) of the marked time points (*p* < 0.05, Wilcoxon signed-rank test).

**Figure 4:**
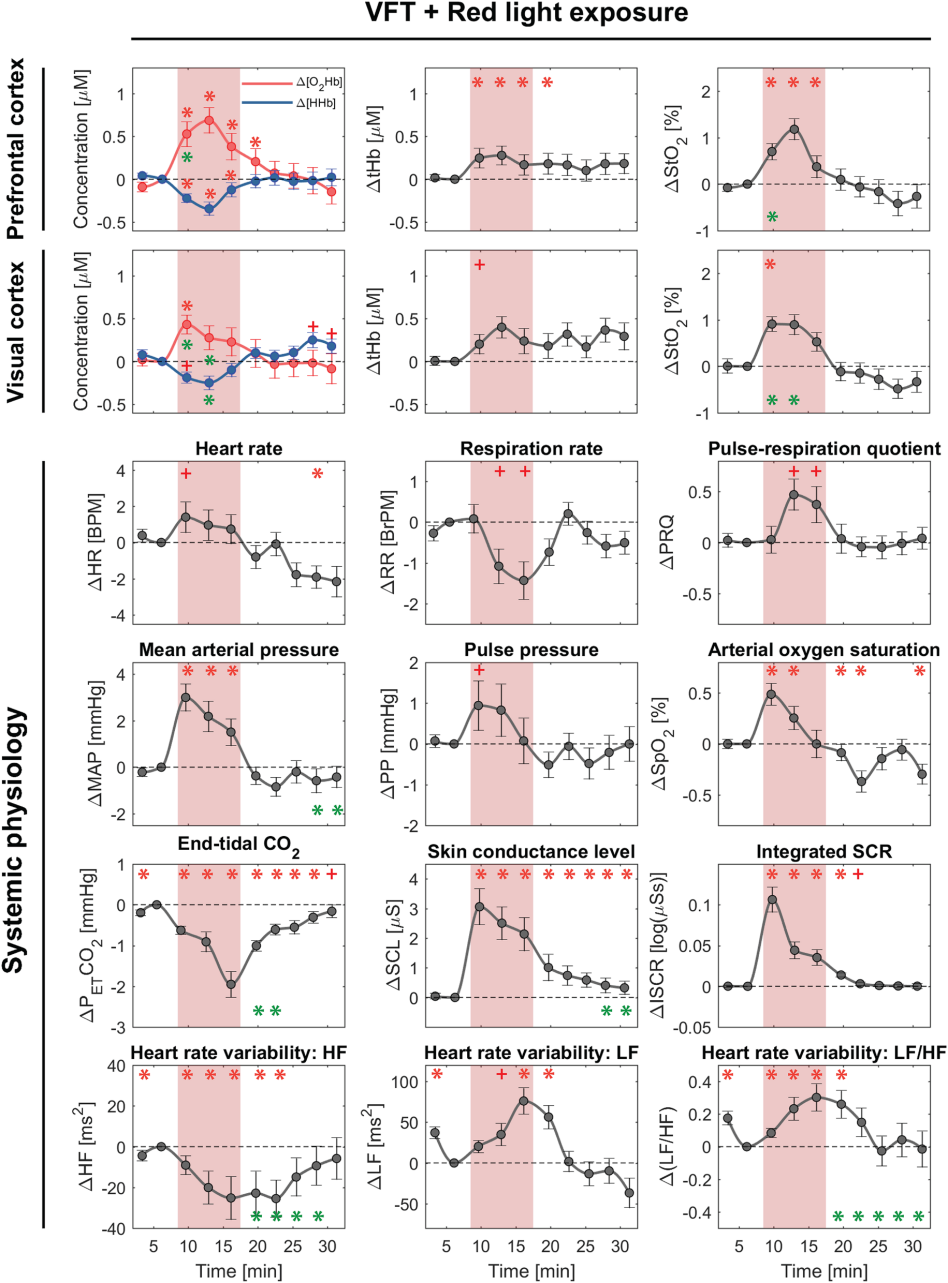
Block-averaged (group-level) changes in cerebral hemodynamics/oxygenation and systemic physiology (median ± SEM) evoked by red light exposure and VFT. The red shaded areas represent time intervals during the task/stimulation period. The time series are sub-divided into ten periods (3 min each). Then, they are normalized to the last time period of the baseline (TP2). Red symbols indicate a significant change of the marked time point with respect to baseline (asterisk *: proved by both FDR-corrected and uncorrected *p* values; plus + : only uncorrected *p* value, *p* < 0.05, Wilcoxon signed-rank test). Green asterisks present a color-difference (blue vs. red) of the marked time points (*p* < 0.05, Wilcoxon signed-rank test).

Figures 3 and 4 depict block-averaged changes in cerebral hemodynamics, oxygenation, and systemic physiology evoked by CLE (blue vs. red) and VFT. Independent of the color, CLE in combination with VFT elicited responses in the PFC and caused significant changes in systemic physiological parameters including MAP, P_ET_CO_2_, SCL, ISCR, and HRV (LF/HF).

#### Cerebral tissue hemodynamics and oxygenation

Both conditions (blue and red) elicited statistically significant changes in [O_2_Hb] (increase), [HHb] (decrease), [tHb] (increase), and StO_2_ (increase) in the PFC, while only the blue light exposure evoked significant changes in the VC, i.e., [O_2_Hb] (increase), [HHb] (decrease), [tHb] (increase), and StO_2_ (increase), throughout the whole CLE-VFT. In the VC, the red light caused only significant stimulus-evoked changes, i.e., [O_2_Hb] (increase) and StO_2_ (increase), at the onset of the CLE-VFT. Color-dependent effects were observed in [O_2_Hb], [HHb], and StO_2_ in the VC. Blue evoked stronger overall responses in cerebral hemodynamics and oxygenation in the VC. Additionally, higher [O_2_Hb] and StO_2_ changes at the beginning (TP3) of the blue light exposure were observed in the PFC compared to the red light exposure.

#### Systemic physiological activity

The following systemic physiological parameters showed statistically significant changes during the CLE-VFT: MAP, SpO_2_, SCL, ISCR, and LF/HF (increase during blue and red); P_ET_CO_2_ (decrease during blue and red); HR and PP (increase during blue); HF (decrease during red). HR, MAP, and SCL changes were higher during blue light in comparison with red light, although the differences did not reach statistical significance. In addition, blue and red light exposure evoked no statistically significant changes in RR and PRQ after FDR correction. PRQ increased for both conditions, although the increase was less pronounced for the blue light.

Color-dependent changes in the recovery phase (post-light period) of some systemic physiological parameters were noticeable (e.g., P_ET_CO_2_: at the beginning of the recovery phase, SCL: at the end of the recovery phase). Changes in HF and LF/HF were color-dependent throughout the recovery phase.

## Discussion

### No significant difference in cognitive performance between blue and red

The VFT has been widely used as a tool to measure verbal ability and executive control. VFT performance also provides a possible predictor for prospective identification of diseases^24,50^. One of the main goals of this study was to investigate the effects of light of two colors (blue vs. red) on VFT performance. No significant difference in subjects’ performance was found between blue and red light. One possible explanation for this finding is that the attention of the subjects was mainly focused on performing the VFT than actively perceiving and being aware of the CLE. This may have diminished the effect of the specific colored light on performance. The number of correct answers produced by the subjects in both versions of the VFT is in good agreement with Holper et al.^50^ and higher compared to the previous studies^23,25,51^. All aforementioned VFT studies were conducted under normal lighting conditions. For the first time, the light of different colors was employed in our study to investigate its impacts on the subjects’ performance. In addition to CLE, age, education level, physiological state of subjects, or even methodological variabilities may be possible reasons for the difference between studies in terms of VFT task performance. Herrman et al. showed that subjects (*n* = 14, middle to highly educated subjects, age: 31.4 ± 6.8 years) achieved an average of 33.0 ± 11.2 correct responses for the three letters (i.e., A, F, and S; each lasted 60 s) in the VFT^25^. In another study, subjects (*n* = 325, including right-handed, left-handed, and ambidextrous, age: 51–82 years) within 30 s produced 6.1 ± 2.0, and 9.9 ± 2.0 correct words in letter and category VFT, respectively^23^. Additionally, within both conditions (blue vs. red), performance in category fluency was significantly better than in letter fluency. These findings are in line with several studies^23,50,51^. The letter fluency is generally more challenging than the category fluency. In the category fluency, subjects can rely on existing links between related concepts or words, while the links between words in the letter fluency may be weaker or less accessible^24^. In other words, retrieval of a word (e.g., apple) automatically activates semantically associated words (e.g., orange, banana, peach, and mango) in the category fluency version of the VFT. By contrast, in the letter fluency, subjects must restrain the activation of associatively related concepts and employ different retrieval strategies^52,53^. In conclusion, we found a reasonable performance compared to previous studies but no effect of the CLE, which was tested for the first time.

### Females were better VFT performers than males

Consistent with previous studies^23,54^, we found that females generally performed VFT tasks more efficiently than males. This fact has become well-accepted, although some findings are inconsistent^55,56^, where the task performance was equal between males and females. It has been proven that female subjects are better in the performance of verbal tasks, while male subjects are more successful in visual-spatial tasks^57,58^. The reason for sex differences at a behavioral level may be attributed to dissimilarities in the structure of the language-related cortex and the cerebral organization of language function^59^. It is known that language functions are more lateralized in males (i.e., to the left inferior frontal gyrus regions) than in females^60^. However, Weiss et al. proposed that such a difference in executive speech tasks is related to different processing strategies for lexical verbal fluency rather than sex-related hemispheric organization^59^. Additionally, the reasons for the significant difference between females and males in VFT performance during the blue light condition may be attributed to different color preferences and color perception between females and males or even fluctuations in sex hormones. Corticotropin-releasing hormone (CRH) plays key roles in the coordination of the stress response as a potential mediator of sex-related differences^61,62^. The presence of estrogen, a classical female sex hormone, has been proved in the CRH gene^61^. Therefore, sex differences at a behavioral performance may also be due to the role of estrogen in mediating stress response. It was demonstrated that a stressful experience caused a female’s later ability to attain certain types of new memories and was dependent on changing levels of estrogen^63^. Shors and Leuner showed that performance of the classically conditioned eyeblink response was poor in the presence of very low and very high estrogen, whereas in the presence of moderate estrogen, performance was optimal^63^. It was also shown that females were better performers on spatial tests in low levels of estrogen and on articulatory-verbal tests in high levels of estrogen^64^. Coupled with the knowledge that estrogens in females and androgens in males are generally positively associated with the performance in verbal and spatial tasks, respectively, for our results, this implies that the ability of estrogen to enhance verbal performance in females can be taken as evidence for sex differences in cognitive functioning underlying the VFT.

### Cerebral hemodynamics and oxygenation

In this study, we used FD-NIRS to investigate a mixed-effect of CLE and VFT on neural correlates of cognitive functioning. The FD-NIRS system is able to measure the absolute optical properties, namely the absorption coefficient and the reduced scattering coefficient, and consequently the absolute values of [O_2_Hb], [HHb], [tHb] and StO_2_. In contrast, continuous wave NIRS (CW-NIRS) can only provide information on changes of [O_2_Hb] and [HHb] but cannot determine absolute values^44^. The CW-NIRS technique relies on assumed constant optical properties during the measurement, an assumption not necessarily true in reality^34^. Compared to CW-NIRS, the FD-NIRS technique is less sensitive to physiological noise from the extracerebral tissue compartment^45^. For further, more detailed information on NIRS-based techniques, we refer readers to Scholkmann et al.^28^. In our previous studies, we investigated how cerebral hemodynamics and oxygenation change during different short-term and long-term colored light exposures^13,38,41,65–67^. The impact of the VFT on human brain activity and cerebral perfusion was also investigated in several fNIRS studies^68–71^. We found in the current study that CLE in combination with VFT evoked responses in the PFC, while only blue light leads to significant changes in the VC throughout the CLE-VFT.

### Color-independent prefrontal cortex responses

The PFC is involved in various higher-order cognitive functions, including memory, processing of language, selective attention, and task planning^70,71^. PFC activity can be evaluated by measuring changes in [O_2_Hb], [HHb], [tHb], and StO_2_. Our findings showed that, regardless of the color type, the CLE with a combination of the VFT leads to an increase in [O_2_Hb], [tHb] and StO_2_, and a concurrent decrease in [HHb], which corresponds to the typical pattern of cerebral activation. However, higher [O_2_Hb] and StO_2_ changes at the beginning (TP3) of the blue light exposure were evident compared to the red light.

Several studies using fNIRS have shown increases of [O_2_Hb] and decreases of [HHb] in response to a VFT task^31,32,68,70^. A recent review concluded that cognitive tasks caused the [O_2_Hb] increasing in more than 70% and [HHb] decreasing in 100% of the studies, which reported changes in hemoglobin concentrations in the PFC in response to the VFT or working memory tasks^72^.

Concerning the CLE, we demonstrated in our previous study that short-term blue light led to a significantly different response in [O_2_Hb], [tHb], and StO_2_ in the PFC compared to red and green light^38^. A significant increase in StO_2_ was indicated in the left PFC during blue but not red light in a long-term CLE study carried out by Weinzirl et al.^66^. In another study where we had investigated long-term CLE, cerebral hemodynamics and oxygenation in the PFC were significantly different for yellow compared to red and blue, but not between red and blue^41^.

We showed that CLE accompanied by the VFT elicited significant changes in cerebral hemodynamics and oxygenation in the PFC. In parallel research performed with this study, we found that CLE alone did not significantly affect cerebral perfusion in the PFC (data not shown). Therefore, it seems that the impact of VFT was more prominent compared to CLE and the stimulating effect of CLE is low or even decreases when the brain is already involved in a challenging VFT task.

### Color-dependent visual cortex responses

Compared to red light, we showed that blue light exposure evoked stronger overall responses in cerebral hemodynamics and oxygenation in the VC. In the literature, there are a few fNIRS reports investigating the effect of color and colored light on cerebral responses in the VC^13,38,73–75^. Even though Liu and Hong^75^ showed that the left VC is more active during the blue-color stimulus, Scholkmann et al.^38^, from our group, observed that the magnitude of the hemodynamic responses in the VC was independent of color. In another fNIRS study, the authors found a significant increase in hemodynamics during the between-category (blue vs. green) changes, but not during the within-category (two different shades of green) changes^73^. The visual system response to blue light might be a marker for central nervous system dopamine tone^76^, reported in an fMRI study. It was also proposed in other neuroimaging studies that colors in different categories are differently represented in the VC^77,78^.

### Systemic physiology responses

A higher increase in HR during exposure to blue light is in line with the research performed by Cajochen et al.^79^ (*n* = 10 male subjects, age: 25.9 ± 3.8 years, exposure to 2 h of monochromatic lights in the evening). Contrary, a decrease was observed in one study under blue light in subjects with closed eyes (*n* = 7, age range: 23–55 years, exposure to 10 min color light panels (blue vs. red), illuminance: 140 lx, distance: 40 cm)^80^. All other studies presented no dependence of HR on CLE in sitting subjects^81–83^. The increase in HR during the blue light condition may be attributed to the autonomic nervous system (ANS) responding to blue light with an increase in sympathetic tone, i.e., a response that is predominantly susceptible to short-wavelength light^79^.

In line with other research^83,84^, in our study, colored light had no distinctive effects on RR based on FDR-corrected *p* values. This could be due to the large inter-subject variation caused by subjects having different RR responses. The absolute RR values measured in our previous study for healthy subjects at rest were within a large range (6.9–27.1 breaths per minute)^44^.

The PRQ is a useful and unitless parameter to attain the overall state of human physiology^46^. This parameter is calculated as HR divided by RR and its resting-state distribution has a peak at ~ 4^85^. Although PRQ increase was more pronounced for the red light, we observed no statistically significant changes (FDR-corrected *p* values) in response to both conditions, which is in accordance with the literature^84^. In our previous study investigating short-term CLE effects with blue light, a decrease in PRQ was found^38^.

We also found an increased MAP for both conditions and an increased PP only for blue light exposure. Increased diastolic blood pressure was reported for the light of higher color temperatures (i.e., 7500 °K vs. 3000 °K and 5000 °K) at high-intensity levels (> 320 lx)^86^. This indicates that light with an increased fraction of blue light increases blood pressure. In another study, MAP increased for blue light, but not for red at a high intensity (> 250 lx)^87^.

The decrease in P_ET_CO_2_ during the CLE and VFT tasks is in line with the findings of Scholkmann et al.^40^. In that study, the effect of different speech tasks on P_ET_CO_2_ and cerebral hemodynamics and oxygenation was investigated. During all tasks, P_ET_CO_2_ was reduced with the strongest decrease during the alliteration task (~ 9 mmHg) and the smallest during the mental arithmetic task (~ 3 mmHg). In the present study, the P_ET_CO_2_ decrease was less than 2 mmHg for both blue and red light. In summary, typical patterns of the fNIRS data and P_ET_CO_2_ caused by a combination of the CLE and VFT are comparable to the mental arithmetic task of the aforementioned study. It is noteworthy that in the current study, the P_ET_CO_2_ decrease constitutes only weak hypocapnia induced by hyperventilation. In this case, the vasodilation caused by brain activity outweighed the vasoconstriction caused by hypocapnia.

Electrodermal activity (EDA) and HRV are two commonly used psychophysiological stress measures^88^. EDA reflects the variation of the electrical properties of the skin in response to sweat secretion^48,49^. In our study, both EDA parameters were significantly increased during blue and red light exposure. The increase in EDA is related to various factors such as mental stress or pain, owing to the stimulation of the sympathetic nervous system. Several studies have shown increasing of EDA parameters during mental load and cognitive stress, compared to baseline measurements^89–91^. In parallel research carried out with this study, we found that CLE alone (without VFT) affected EDA (data not shown; ΔSCL: SCL_CLE_ − SCL_base_; Blue: 0.04 ± 0.08 µS; Red: 0.05 ± 0.07 µS) much lower than the CLE-VFT (this study; Blue: 3.08 ± 0.38 µS; Red: 2.98 ± 0.55 µS). Therefore, we conclude that the effect of the VFT on EDA changes was more noticeable than the CLE. In our previous study, we showed that short-term blue, red, and green light (without any cognitive tasks) triggered changes in SCL with large intersubject variability and only a marginal change in the group-average^38^. Other studies showed an increase in EDA under short-term (1 min) red light^83^ and a decrease under long-term (20 min) blue, orange, and green light exposure^92^.

HRV is an index of the ANS, providing a measure of ANS through parasympathetic and sympathetic modulation of cardiac function^93^. It is known that the LF component of HRV is modulated by both the parasympathetic nervous system (PNS) and the sympathetic nervous system (SNS), while HF is mainly controlled by the PNS^94^. Although it is widely believed that the LF/HF ratio reflects the sympathovagal balance, Billman showed that this assumption is not accurate and greatly oversimplifies the complex non-linear relations between the sympathetic and the parasympathetic divisions of the ANS^95^. In line with this new statement, von Rosenberg et al. proposed a simultaneous consideration of the LF and HF within a 2D scatter diagram, improving the discrimination ability in the physical and mental stress analysis^96^. HRV is considered as a marker of stress. In both conditions, we found a decrease of the HF and an increase of the LF during the CLE-VFT, although only the HF was significantly decreased during red light exposure. An increased LF and a decreased HF observed in both conditions are attributed to the mental stress that subjects experienced during VFT tasks^94,97^. This emphasizes once again the point that the influence of VFT is more prominent compared to CLE during the CLE-VFT and the stimulating effect of CLE is low or even decreases when the brain is already involved in a challenging VFT task. In line with the research carried out by Posada-Quintero and Chon^98^, we found an increase in the LF component of HRV as well as the EDA in the presence of stressors (VFT tasks), which are known to be controlled by the SNS. In several studies, red light decreases HF power^84,99,100^. Even though blue light caused no significant HF changes in our study, an increase in the HF was observed due to blue light in other studies^81,101,102^. We did not observe any significant difference between red and blue during the CLE. However, changes in HF and LF/HF were interestingly color-dependent throughout the recovery phase. This may be indicative that brief exposure to colored light has effects on HRV parameters that may persist for at least 15 min after cessation of the light. Higher HF changes and lower LF/HF ratio after blue light compared to red light may indicate an increase of the parasympathetic response during the recovery phase after blue light exposure. In other words, higher HF changes accompanied by insignificant changes in the LF after blue in comparison with red represent a more relaxed state for this condition based on the 2D scatter diagram proposed by von Rosenberg et al.^96^.

### Limitations

The study has the following limitations: (1) The size of the sample was calculated with power analysis to detect substantial effects (effect size: *d* = 0.59) at a *p* < 0.05 and a power of > 0.8. A large number of subjects may have shown further physiological or behavioral color-dependent responses. But, these effects would have been small and probably not very relevant. (2) Only blue and red light were analyzed. Different colors may have other effects. (3) The intensity of the CLE may evoke different effects. We selected a relatively high intensity (120 lx) that was the same for both colors. (4) The ISS optodes do not yet cover the entire head, and hence not the complete brain was analysed. (5) Depending on the nature of the task, different colored lights might affect cognitive tasks differently. It has been revealed that red improves performance on a detailed-oriented task, while blue enhances performance on a creative task^103^. Therefore, it would be worthwhile to include other cognitive tasks in future studies. (6) The VFT protocol can be simplified for future studies. The control task, which normally does not evoke brain activation and may reduce the effect, can be removed from the VFT protocol.

## Conclusions and outlook

In conclusion, our study is the first employing SPA-fNIRS to investigate a mixed-effect of CLE and VFT on cerebral hemodynamics, oxygenation, and systemic physiology. Our new approach (SPA-fNIRS) enabled to understand the interaction of cerebral and systemic parameters. No significant difference in the subjects’ VFT performance was found between blue and red light exposure, while sex and type of VFT (category versus letter) affected the subjects’ performance significantly. Overall, blue light exposure evoked stronger responses in cerebral hemodynamics and oxygenation in the VC. We found several color-independent changes in cerebral and physiological signals due to the VFT. Moreover, significant differences between red and blue light exposure were observed in the recovery phase (post-exposure period) of systemic physiological parameters (namely, P_ET_CO_2_, SCL, HF, and LF/HF ratio). Therefore, it is essential to consider the relatively long-lasting (15 min) effects of CLE in humans. This underlines the importance of considering the persistent influence of colored light on brain function, cognition, and systemic physiology in everyday life.

In this study, SPA-fNIRS was used to assess a mixed-effect of CLE and VFT on human physiology. The results imply that the reaction of the brain and the systemic physiology is different or in some cases similar, depending on the physiological parameter and color of the light (with equal perceived brightness). In our previous study, with SPA-fNIRS, we were also able to demonstrate that MAP and SpO_2_ were positively correlated with [O_2_Hb] at the PFC during the CLE-VFT^45^. Therefore, SPA-fNIRS should become a standard approach for fNIRS studies to enable a comprehensive understanding and the correct interpretation of changes in cerebral hemodynamics and oxygenation. This tool enables to understand the underlying reasons for a variety of stimulus-evoked changes and cognitive task performances.

Our findings contribute to a better understanding of CLE effects on human physiology. Although no significant difference in the performance of VFT tasks was found between blue and red light, the physiological color-dependent responses are potentially of high relevance in the process of choosing colors and colored lights for related objectives (e.g., room lighting and workplaces). Specifically, our findings about a mixed-effect of CLE and VFT on human physiology offer a broad range of implications for educational purposes and facilitate a better responding of the following questions: *Do colored lights (or colors) play a role in enhancing cognitive task performances as well as learning and nurturing concepts? What colored light improves motivation and creativity in the workplace?* Additionally, since exposure to colored light expeditiously increases in our modern society due to modern light sources such as screens and LEDs, investigation of colored light and its impact on human physiology are of rising interest. Especially at the present time, when students have to do their homework using smartphones and tablets, known as modern light-emitting devices, more than before due to the Covid-19 pandemic, another question might be raised: *What desktop background color do we pick for educational facilities?* The results of this research are expected to facilitate a better understanding of the CLE effects on the underlying neuroscientific mechanisms in the brain and body, which in turn would pave the way for safe and advantageous applications of colored light in daily life and even therapeutic settings. Moreover, in a society that is rapidly exposed to new and increasing lighting, it is expected that the findings of this research are being relevant and beneficial for the scientific community, medical professionals and the society.
